# New Candidates for Autism/Intellectual Disability Identified by Whole-Exome Sequencing

**DOI:** 10.3390/ijms222413439

**Published:** 2021-12-14

**Authors:** Lucia Pia Bruno, Gabriella Doddato, Floriana Valentino, Margherita Baldassarri, Rossella Tita, Chiara Fallerini, Mirella Bruttini, Caterina Lo Rizzo, Maria Antonietta Mencarelli, Francesca Mari, Anna Maria Pinto, Francesca Fava, Alessandra Fabbiani, Vittoria Lamacchia, Anna Carrer, Valentina Caputo, Stefania Granata, Elisa Benetti, Kristina Zguro, Simone Furini, Alessandra Renieri, Francesca Ariani

**Affiliations:** 1 Medical Genetics, University of Siena, 53100 Siena, Italy; lucia.bruno@dbm.unisi.it (L.P.B.); gabriella.doddato@dbm.unisi.it (G.D.); floriana.valentino@dbm.unisi.it (F.V.); margherita.baldassarri@dbm.unisi.it (M.B.); chiara.fallerini@dbm.unisi.it (C.F.); mirella.bruttini@dbm.unisi.it (M.B.); francesca.mari@dbm.unisi.it (F.M.); francesca.fava@dbm.unisi.it (F.F.); alessandra.fabbiani@dbm.unisi.it (A.F.); vittoria.lamacchia@dbm.unisi.it (V.L.); anna.carrer@dbm.unisi.it (A.C.); valentina.caputo@dbm.unisi.it (V.C.); stefania.granata@dbm.unisi.it (S.G.); alessandra.renieri@dbm.unisi.it (A.R.); 2 Med Biotech Hub and Competence Center, Department of Medical Biotechnologies, University of Siena, 53100 Siena, Italy; elisa.benetti@dbm.unisi.it (E.B.); kristina.zguro@student.unisi.it (K.Z.); simone.furini@dbm.unisi.it (S.F.); 3 Genetica Medica, Azienda Ospedaliera Universitaria Senese, 53100 Siena, Italy; rossella.tita@dbm.unisi.it (R.T.); lorizzo2@unisi.it (C.L.R.); mariaantonietta.mencarelli@dbm.unisi.it (M.A.M.); annamaria.pinto@dbm.unisi.it (A.M.P.)

**Keywords:** autism, intellectual disability, whole-exome sequencing

## Abstract

Intellectual disability (ID) is characterized by impairments in the cognitive processes and in the tasks of daily life. It encompasses a clinically and genetically heterogeneous group of neurodevelopmental disorders often associated with autism spectrum disorder (ASD). Social and communication abilities are strongly compromised in ASD. The prevalence of ID/ASD is 1–3%, and approximately 30% of the patients remain without a molecular diagnosis. Considering the extreme genetic locus heterogeneity, next-generation sequencing approaches have provided powerful tools for candidate gene identification. Molecular diagnosis is crucial to improve outcome, prevent complications, and hopefully start a therapeutic approach. Here, we performed parent–offspring trio whole-exome sequencing (WES) in a cohort of 60 mostly syndromic ID/ASD patients and we detected 8 pathogenic variants in genes already known to be associated with ID/ASD (*SYNGAP1*, *SMAD6*, *PACS1*, *SHANK3*, *KMT2A*, *KCNQ2*, *ACTB*, and *POGZ)*. We found four de novo disruptive variants of four novel candidate ASD/ID genes: *MBP*, *PCDHA1*, *PCDH15*, *PDPR*. We additionally selected via bioinformatic tools many variants in unknown genes that alone or in combination can contribute to the phenotype. In conclusion, our data confirm the efficacy of WES in detecting pathogenic variants of known and novel ID/ASD genes.

## 1. Introduction

Intellectual disability (ID), previously known as “mental retardation”, represents a major public health problem [1]. ID is a condition characterized by below-average intellectual functioning (IQ < 70) together with significant limitations in adaptive functioning [2]. ID can be “isolated” or “syndromic” when peculiar facies, typical clinical traits, and/or growth abnormalities are documented [3,4]. Often, a coexistence of ID and autism spectrum disorder (ASD) arises, with 70% of ASD patients also showing ID, and 40% of ID patients displaying ASD [4,5]. ASD encompasses neurodevelopmental conditions characterized by deficient social interactions, poor or absent communication, repetitive behaviors, and apparently limited interests [3,6]. ASD and ID affect about 1–3% of the general population [7,8]. Genetic factors, fetal intrauterine environment, and external environmental factors play important roles in ID and ASD [9,10].

The genetic basis of ID and ASD is deeply heterogeneous, implicating more than 2000 OMIM genes, in turn, involved in different pathways and biological processes, such as those regulating synaptic plasticity, chromatin remodeling, gene transcription, and protein degradation [11,12]. Combining clinical and molecular diagnosis is fundamental to deepen our knowledge of the pathogenic mechanisms underlying these medical conditions and to develop personalized treatments [13]. Single-nucleotide variants (SNVs), indels, and copy number variations (CNVs) have been identified as key variant types causing ID/ASD [7,14].

Up to 50% of the ID/ASD patients remain without a molecular diagnosis [15]. Next-generation sequencing (NGS) technologies have greatly improved the chance of identifying known as well as novel responsible genes [15,16,17]. The use of Whole-Exome Sequencing (WES) together with CNVs analysis can identify a pathogenic variant in about 30% of patients [17]. The current diagnostic yields suggest the application of WES as a routine first-tier diagnostic test permitting an early diagnosis for ID/ASD patients, with improvement in terms of the quality of life of the affected families [18,19].

In the present study, WES was performed for a total of 60 trios with a diagnosis of an ASD/ID-related phenotype. We identified pathogenic variants in already known ID/ASD genes in eight families. We found four new candidate genes with de novo truncating variants (three frameshift deletions/duplications and one nonsense variant), i.e., *MBP, PCDHA1*, *PCDH15*, *PDPR*. Among the remaining cases, we selected missense variants for which bioinformatic tools suggested pathogenicity. We found that these unknown rare variants, alone or in combination with each other, contributed to the phenotype. In conclusion, our data confirm the efficacy of WES in detecting pathogenic variants in known and novel ID/ASD genes

## 2. Results

### 2.1. Study Cohort

In the present study, we enrolled 60 proband–parent trios with a diagnosis of ASD/ID-related phenotype. Probands were a total of 15 females and 45 males with a mean age of 10 years. Patients diagnosed with ID were 19, one patient had high-functioning ASD, and 40 patients had ASD and ID. Of the 60 patients, 12% presented epilepsy, 80% language delay, 66% psychomotor delay, and 73% craniofacial dysmorphisms. The clinical description of the patients is reported in Table S1.

### 2.2. WES and Variants Pathogenicity Assessment

WES was performed for 60 proband–parent trios, and a mean coverage of 94 reads for targeted sequenced regions was obtained. Patients were previously analyzed by array-CGH and were all negative. Pathogenic variants were selected according to variant frequency and category, co-segregation with the disease, literature data, and database classification (ClinVar database).

We selected novel candidate genes starting from truncating variants that could be assumed to disrupt gene function (Table 1). We found four cases with de novo truncating variants (three frameshift deletions/duplications and one nonsense variant) in genes not traditionally associated with ID/ASD: *MBP*, *PCDHA1*, *PCDH15*, and *PDPR* (Table 1) (ST2). For missense variants CADD phred values higher than 20 suggested pathogenicity (Table S3). We also performed structural predictions through the HOPE tool (Table S2). Potential splicing impact was predicted for the variants in the unknown genes *TYRO3*, *OPN4*, *CBX3*. We found eight pathogenic variants (two frameshift deletions/duplications, three nonsense, and three reported missense variants) in genes already known to be associated with ID/ASD: *SYNGAP1*, *SMAD6*, *PACS1*, *SHANK3*, *KMT2A*, *KCNQ2*, *ACTB*, and *POGZ* (Table). Some variants were reported in the database (ClinVar database) or previously described in the scientific literature (Table 1, Table 2 and Table 3). VUS were detected in 28 novel genes and in 10 known ID/ASD genes (Table S3). VUS were found alone or in combination with each other (Table S3).

### 2.3. Clinical Features of Patients with Truncating Variants of ID/ASD Candidate Genes

**Figure 1:**
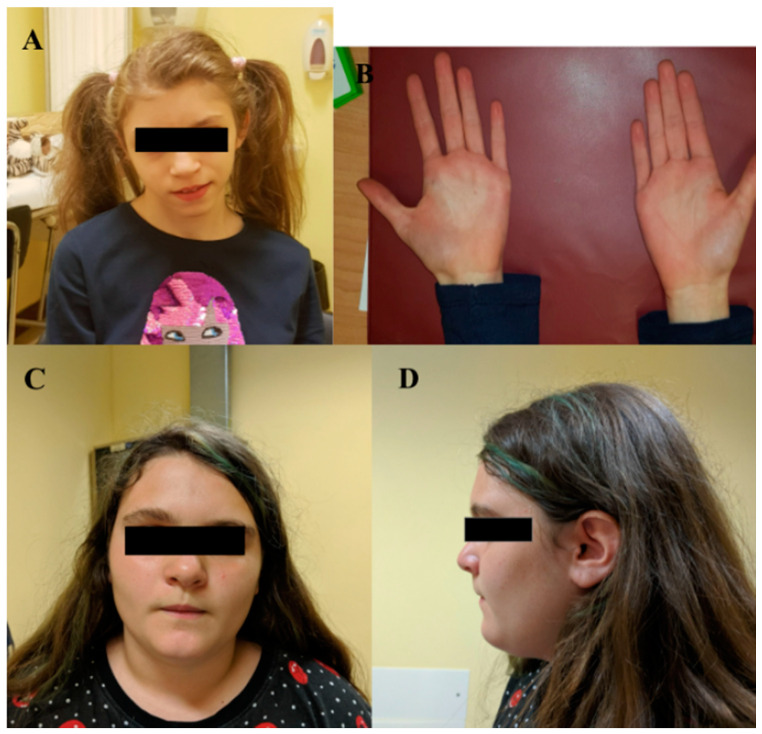

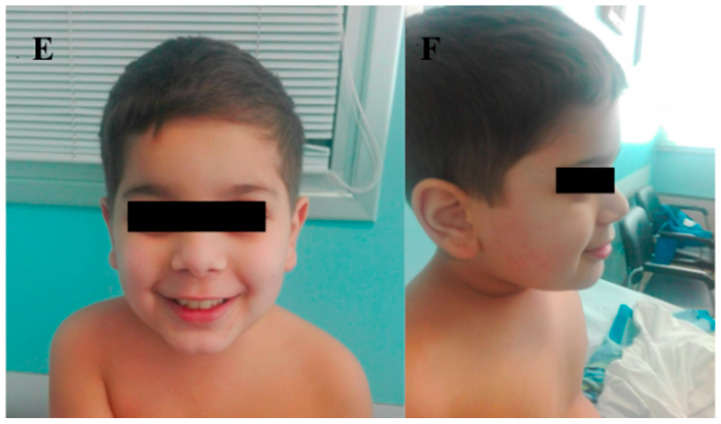
(**A**) Frontal view of proband I (*MBP* gene; p.(Phe46fs*18)) showing triangular facies, prominent ears, thin upper lip, absent eyebrows, broad nasal bridge, bulbous nasal tip, thin and sparse hair, everted lower lip, advanced hairline; (**B**) close-up of the arachnodactyly of the hand of proband I; (**C**) frontal view of proband II (*PCDH15* gene; p.(Lys1859Asnfs*)) displaying square-shaped face, deep-set eyes, bilateral underfolded helix, short and stocky neck; (**D**) lateral view of proband II; (**E**) frontal view of proband IV (*PDPR* gene; p.(Gln276*)) showing big and deep-set eyes, wide nasal tip, thin upper lip, chin dimple and macrodontia; (**F**) lateral view of proband IV.

Four patients showed rare de novo truncating variants of the novel candidate genes *MBP*, *PCDHA1*, *PCDH15*, *PDPR*. The clinical findings of each patient are described in Table 3 and Table S1. Patient I was a 15-year-old female child with a diagnosis of ID. Pregnancy was complicated by intrauterine growth retardation. However, her karyotype was normal. She was born at 38 weeks of gestation. The growth parameters at birth were: length of 46 cm (3–10° percentile), weight of 2300 g (10–25° percentile), and Occipital Frontal Circumference (OFC) of 34 cm (50° percentile). At birth, she was diagnosed with interatrial septal defect (DIA) and interventricular septal defect (DIV). Since the first months of life, she suffered from esophageal reflux and feeding difficulties. She started independent walking at the age of 19 months, but her speech development was severely delayed. Sphincter control was acquired at 3 years. Recurrent airway infections emerged during childhood. Physical examination at the age of 13 years showed height of 144.5 cm (3–10° percentile), weight of 35 kg (3–10° percentile), and head circumference of 50.5 cm (−2.4 SD). She presented a triangular face with low anterior hairline, broad nasal bridge with bulbous nasal tip, M-shaped upper lip, and everted lower lip. Arachnodactyly of the hands and feet was noticed (Figure 1A,B). She was attending school with support and had poor reading and writing skills. She received physiotherapy and psychotherapy and showed good interaction with peers. Parents reported hand stereotypies, episodes of unprovoked laughter, hyperactivity, and sleep disorder. Her array CGH analysis did not reveal any chromosomal aberrations. WES analysis was thus performed, and we detected a de novo truncating variant c.138del (p.(Phe46fs*18)) of the *MBP* gene.

Patient II, a girl (14 years of age) diagnosed with ID and affected by epilepsy, carried a frameshift de novo variant c.5573_5576 (p.(Lys1859Asnfs * 2)) of *PCDH15* and a de novo splicing variant c.1074–11G > A of *OPN4*. She was attending school with a support teacher. She had facial dysmorphisms such as a square-shaped face, deeply set eyes, bilateral underfolded helix, and short and stocky neck (Figure 1C,D). She was born at term after a pregnancy with gestational diabetes. Parameters at birth were: length, 48 cm (25–50° percentile), weight, 2930 g (10–25° percentile), and APGAR, 9–10. No suction difficulties were observed. At 16 months of age, she started to present seizures for which she is still on therapy. She acquired autonomous deambulation at 18 months, and language and psychomotor delay were reported.

Patient III was a 9-year-old male presenting with ADHD (Attention-Deficit/Hyperactivity Disorder) and behavior disorder. Both parents are healthy, without a family history of neurodevelopmental disorder. He was diagnosed with ID and ASD. He was born at term, without any problems during pregnancy. He started walking and pronounced the first words at 1 year of age. Cerebral Magnetic Resonance Imaging (MRI) revealed reduced white matter, hypoplasia of vermis and trunk, tortuous optic nerves and vertebral arteries. EEG showed left-sided centro-temporal epileptic elements. Physical examination at the age of 9 years showed height of 136 cm (66° percentile), weight of 33 kg (72° percentile), and OFC of 54 cm (86° percentile). He presented simplified auricles and no further dysmorphic signs. Via WES we found a de novo frameshift mutation c.670_673dup (p.(Thr225Argfs*4)) in the *PCDHA1* gene.

We visited patient IV when he was 8 years and 10 months old. He was born at term by cesarean section. During the pregnancy, her mother suffered from a cytomegalovirus infection. His perinatal and postnatal period were normal. He pronounced his first words at the age of 12 months and walked independently at 13 months. Sphincter control was acquired at 38 months. He presented difficulty in social interactions, with a tendency to isolation. The patient showed difficulties in acquiring reading–writing skills and received psychomotor and speech therapy. He received a diagnosis of ID. Clinical examination revealed height of 125 cm (10–25° percentile), weight of 31 kg (50–75° percentile), and head circumference of 54 cm (75–90° percentile). His dysmorphic facial features included deeply set eyes, wide nasal tip, thin upper lip, chin dimple, and macrodontia (Figure 1E,F). Furthermore, hypochromic spots were observed on the back and upper limbs. A de novo stop-gain variant c.826C > T (p.(Gln276*)) of *PDPR* was revealed by WES.

## 3. Discussion

Identifying the etiology of ASD and ID poses an arduous challenge due to a relevant clinical heterogeneity and a high genetic heterogeneity of these medical conditions, even across single families [23,24]. WES demonstrated to effectively detect novel candidate genes potentially associated with these neurodevelopmental disorders [3]. In the present study, we performed WES in a cohort of 60 trios with a diagnosis of ASD/ID-related phenotype and we detected 8 pathogenic variants in genes already known to be associated with ID/ASD (*SYNGAP1*, *SMAD6*, *PACS1*, *SHANK3*, *KMT2A*, *KCNQ2*, *ACTB*, and *POGZ)*. We also identified four de novo disrupting variants in the following candidate genes: *MBP, PCDHA1, PCDH15,* and *PDPR*.

*MBP* encodes the principal constituent of myelin, needed for the maintenance of the compact multilamellar membrane structure of its mature form [25,26]. *MBP* is expressed in oligodendrocytes in the central nervous system and in Schwann cells in the peripheral nervous system [26]. Mice with mutations in the *MBP* gene developed a decreased myelination of the central nervous system, with tremors and convulsions, progressively leading to an early death [27]. A caudal-to-rostral gradient of transcription for *MBP* has been observed in the developing human brain, reflecting the process of myelination [28]. A variant in the *MBP* gene was found in patient I, who presented hand stereotypies, hyperactivity, sleep disorder, and speech delay.

The *PCDHA* gene cluster consists of 14 tandemly arranged genes that are expressed in the vertebrate brain and encode diverse membrane proteins involved in axonal projection, learning, and memory [29,30].

*PCDHA1* was found mutated in a patient with ADHD, language and psychomotor delay, simplified auricles, and abnormal cerebral structures. Among the protocadherin superfamily members, *PCDH15* pathogenic variants cause Usher syndrome following an autosomal recessive inheritance pattern [31,32,33]. Although primarily recognized as a disease associated with deafness and blindness, more than 20% of Usher syndrome patients display psychiatric symptoms, and comorbidities of Usher syndrome with various mental illnesses are well documented [34,35,36,37]. Moreover, rare heterozygous SNVs in PCDH15 were detected in a patient with schizophrenia and ASD [38]. Importantly, a 386 kb deletion in 10q21.1, including the first three exons of *PCDH15*, was reported in a subject with ASD [39]. We found a *PCDH15* frameshift variant in a patient with ID, epilepsy, and language, and psychomotor delay and we hypothesized that, in part, it might contribute to the phenotype.

The pyruvate dehydrogenase phosphatase regulatory subunit (*PDPR*) is variably expressed in the brain, with the highest levels in the corpus callosum and in the cerebellum [40,41]. *PDPR* has been recently proposed as a novel candidate gene for ID. A missense variant in homozygosity, in fact, was found in a patient with global developmental delay, Joubert-like symptoms, and MRI findings [42]. Here, we propose that the de novo *PDPR* stop-gain variant might contribute to the language delay and behavior disorder observed in patient IV.

Finally, we found VUS in ID/ASD known genes (*BRWD3*, *CNOT1*, *DNMT3A*, *FGF13*, *HUWE1*, *KMT2B*, *NLGN4X*, *PHF8*, *TAF1*, *DDX3X*) and in novel genes (*RGPD4*, *RIN3*, *SBNO2*, *RBMS1*, *SORBS1*, *CHRFAM7A*, *RSF1*, *AGPAT5*, *GALR3*, *NRXN2*, *TYRO3*, *MAP3K10*, *SLC7A8*, *LONP1*, *CBX3*, *DCAF11*, *GINS2*, *SLC8A1*, *CDC7*, *IPO7*, *POTEH*, *MAP3K5*, *SORBS1*, *POU3F2*, *KANSL3*, *IQSEC3*). These variants have been found alone or in combination. The association between de novo SNVs and ASD/ID has been demonstrated by different studies, firstly for disruptive variants [43]. Recently, an enrichment of multiple de novo variants in various genes found via large-scale exome sequencing has suggested the involvement of an oligogenic model in patients with ASD [44]. Our findings are in agreement with the theory of a multi-hit model in the pathogenesis of ASD and ID [45].

In conclusion, we confirmed that WES offers expanded diagnostic options for patients with ID/ASD who resulted negative to array-CGH analysis. However, these genes are candidates, and additional studies are required to assess their role in neurodevelopment, to determine if they are related to abnormal phenotypes according to a monogenic model or a polygenic model and if modifier genes and environmental factors can alter the associated phenotypes [46].

## 4. Materials and Methods

### 4.1. Selection of Patients and DNA Samples Preparation

Genetic counseling was carried out to evaluate each patient’s personal and familial history. Parents provided and signed a written informed consent at the Medical Genetics department of the University of Siena for exome sequencing analysis, clinical data usage, and the use of DNA samples from the tested individuals for both research and diagnosis purposes. We analyzed 60 patients affected by ID and ID/ASD (19 with ID, 40 with ID and ASD, and 1 with isolated ASD) recruited from January 2019 until the end of August 2021.

Genomic DNA from the probands and parents was isolated from EDTA peripheral blood samples using MagCore HF16 (Diatech Lab Line, Jesi, Ancona, Italy) according to the manufacturer’s instructions.

### 4.2. Whole-Exome Sequencing

Sample preparation was performed following the “Illumina DNA prep with enrichment” manufacturer’s protocol. This protocol involves the use of a bead-based transposome complex to perform tagmentation, a process that fragments the genomic DNA and then tags it with adapter sequences in one step. After saturation with input DNA, the bead-based transposome complex fragments a set number of DNA molecules. This fragmentation provides flexibility to use a wide DNA input range to generate normalized libraries with a consistent tight fragment size distribution. Then, a limited-cycle PCR adds adapter sequences to the ends of a DNA fragment. A subsequent target enrichment workflow is applied. Following pooling, the double-stranded DNA libraries are denatured and biotinylated. Illumina Exome Panel v1.2 (CEX) probes are hybridized to the denatured library fragments. Then Streptavidin Magnetic Beads (SMB) capture the targeted library fragments within the regions of interest. The indexed libraries are eluted from the beads and further amplified before sequencing. Whole-exome sequencing analysis was performed on the Illumina NovaSeq6000 System (Illumina San Diego, CA, USA) according to the NovaSeq6000 System Guide. Reads were mapped against the hg19 reference genome using the Burrow–Wheeler aligner (BWA) [47]. Variant calling was obtained using an in-house pipeline which takes advantage of the GATK Best Practices workflow [48].

### 4.3. Filtering and Variant Prioritization

All variants were screened according to frequency, location, mutation category, literature, and mutation database data (ClinVar database, LOVD database, HGMD database). Polymorphisms (minor allele frequency, MAF < 0.01) were excluded, and synonymous variants were assumed to be benign or likely benign. Missense variants were predicted to be damaging by CADD-Phred prediction tools for functional effect prediction. Frameshift, stop-gain, and splice site variants were prioritized as pathogenic. A prediction of damage for the unreported missense variants in the new candidate genes came from HOPE and Phyre2 tools. The potential impact of the variants on splicing was evaluated using Alamut^®^ Visual software—version 2.11-0 (Interac-tive Biosoftware, Rouen, France), which employs five different algorithms: SpliceSiteFinder-like, MaxEntScan, NNSPLICE, GeneSplicer, and HumanSplicingFinder.

The following public databases were used for the interpretation of the variants: ClinVar (https://www.ncbi.nlm.nih.gov/clinvar/, accessed 26 November 2021), LOVD (https://databases.lovd.nl/shared/genes, accessed 26 November 2021), Human Genome Mutation Database (HGMD, http://www.hgmd.cf.ac.uk/ac/index.php3, accessed 26 November 2021).

### 4.4. Sanger Sequencing

Pathogenic variants in new candidate genes were confirmed by Sanger sequencing. DNA samples were sequenced using the PE Big Dye Terminator Cycle Sequencing Kit on an ABI Prism 3130 analyzer (Applied Biosystems). The data were analyzed using the Sequencher version 4.9 software.

## Figures and Tables

**Table 1:** Molecular information for the four truncating variants of novel ID/ASD candidate genes.

Proband	Gene	Transcript (hg19)	Variant (HGVS)	Protein (HGVS)	MAF (gnomAD All)	MAF (gnomAD NFE)	dbSNP	ClinVar Classification	CADD	Transmission	Origin	Classification
I	*MBP*	NM_001025081.1	c.138del	p.(Phe46Leufs * 18)	NA	NA	NA	NA	NA	Autosomal dominant	De novo	Pathogenic
II	*PCDH15*	NM_033056.3	c.5573_5576dup	p.(Lys1859Asnfs * 2)	0.0068%	0.011%	rs770082088	NA	NA	Autosomal dominant	De novo	Pathogenic
III	*PCDHA1*	NM_018900.3	c.670_673dup	p.(Thr225Argfs * 4)	NA	NA	NA	NA	NA	Autosomal dominant	De novo	Pathogenic
IV	*PDPR*	NM_001322118.1	c.826C > T	p.(Gln276*)	0.00071%	0.0016%	NA	NA	22.7	Autosomal dominant	De novo	Pathogenic

* means change in a stop codon.

**Table 2:** Molecular information for the pathogenic variants of genes already known to be associated with ID/ASD.

Proband	Gene	Transcript (hg19)	Variant (HGVS)	Protein (HGVS)	MAF (gnomAD All)	MAF (gnomADNFE)	dbSNP	ClinVar Classification	CADD	Transmission	Origin	Classification	Reference
VIII	*ACTB*	NM_001101.4	c.583G > A	p.(Glu195Lys)	NA	NA	NA	Likely pathogenic	37	Autosomal dominant	De novo	Likely pathogenic	NA
IX	*KCNQ2*	NM_172107.2	c.628C > T	p.(Arg210Cys)	NA	NA	rs796052626	Pathogenic	27.3	Autosomal dominant	De novo	Pathogenic	[20]
X	*KMT2A*	NM_001197104.1	c.478C > T	p.(Arg160*)	NA	NA	NA	NA	36	Autosomal dominant	De novo	Pathogenic	NA
XI	*PACS1*	NM_018026.3	c.607C > T	p.(Arg203Trp)	0%	NA	rs398123009	Pathogenic	29.4	Autosomal dominant	De novo	Pathogenic	[21]
XII	*POGZ*	NM_145796.3	c.2716C > T	p.(Arg906 *)	NA	NA	rs869312833	Pathogenic	12.48	Autosomal dominant	De novo	Pathogenic	[22]
XIII	*SHANK3*	NM_001080420.1	c.1807_1811del	p.(Val604Leufs * 80)	NA	NA	NA	NA	NA	Autosomal dominant	De novo	Pathogenic	NA
XIV	*SMAD6*	NM_005585.4	c.137dup	p.(Tyr459Leufs * 106)	NA	NA	NA	NA	NA	Autosomal dominant	De novo	Pathogenic	NA
XV	*SYNGAP1*	NM_001130066.1	c.3670C > T	p.(Arg1224 *)	NA	NA	rs869312955	Pathogenic	36	Autosomal dominant	De novo	Pathogenic	[23]

**Table 3:** Genotype–phenotype correlations for the candidate genes.

Proband	Gene	Variant (HGVS)	Protein (HGVS)	A Gender	Age (Years Old)	ID/ASD	Craniofacial Dysmorphisms	Additional Clinical Signs
I	*MBP*	c.138del	p.(Phe46Leufs*18)	F	15	ID	Triangular facies, prominent ears, thin upper lip, absent eyebrows, broad nasal bridge, bulbous nasal tip, thin and sparse hair, everted lower lip, M-shaped upper lip, hairline anteriorly advanced.	Hyperactivity, language delay and aggressiveness, disturbed wake–sleep cycle, arachnodactyly of the hand and feet.
II	*PCDH15*	c.5573_5576dup	p.(Lys1859Asnfs * 2)	F	14	ID	Square-shaped face, deeply set eyes, bilateral underfolded helix, short and stocky neck.	Epilepsy, language and psychomotor delay.
III	*PCDHA1*	c.670_673dup	p.(Thr225Argfs * 4)	M	9	ASD and ID	Simplified auricles	ADHD, vermis and brain stem hypoplasia, tortuous course of the optic nerves, flat feet, hyperlaxity, psychomotor and language delay.
IV	*PDPR*	c.826C > T	p.(Gln276*)	M	11	ID	Deep-set eyes, wide nasal tip, thin upper lip, chin dimple, and macrodontia.	Cognitive impairment, repetitive behaviors, an altered sleep pattern with difficulty in falling asleep, isolationist tendencies, manual stereotypies, hypochromic stains, food selectiveness, language and psychomotor delay.

* means change in a stop codon.

## Data Availability

NGS data has been deposited in publicly accessible repositories. The data can be found here: http://nigdb.cineca.it/.

## Supplementary Materials

The following are available online at https://www.mdpi.com/article/10.3390/ijms222413439/s1.
